# Yerba Loca

**Published:** 1888-09

**Authors:** R. H. L. Bibb

**Affiliations:** Saltillo, Mexico


### YERBA LOCA.

By R. H. L. Bibb, M. D., Saltillo, Mexico.

For Daniel's Texas Medical Journal.

JUDGING from the varied accounts that have recently appeared in a number of secular and professional publications, there is but little consensus of opinion (a less display of knowledge) concerning the medical properties, the therapeutical effects, the toxic qualities, or the botanical characteristics of the so-called "Yerba loca" of Mexico. To furnish data for the correction of these discrepancies, is the object of this paper—an object the writer hopes to be able to accomplish to the reader's satisfaction.

The term "Yerba loca"—sometimes incorrectly written "loco"—is a misapplication of a Spanish noun and adjective, and literally means mad herb; crazy herb; but in its application among Mexicans, it is employed to designate the *Cannabis Indicæ*, the *Datura Stramonium* and the *Datura Fatula*, (especially the two latter) plants which they believe to be capable of producing insanity in the human subject, and which are often used for that purpose by the evil designing and revengeful.

To satisfy himself conclusively, and to eliminate any doubt as to the identity of the "Yerba loca," so-called, the writer took the trouble to procure a number of samples from different portions of the Republic, and in every instance, the specimens furnished have been one variety or other of the Jamestown weed,* called "Toloache," "Yerba hedionda," "Yerba del diablo," "Yerba de los hechiceros," "Manzana espinosa," "Nacázcul," "Toloalzin," and "Traplatl," by the Indians; or of the Indian hemp, known also as "Marihuana,"† "Rosa Maria," "Hachisch," and "Cáñamo Indiano." Frequently, all three have been supplied, accompanied with the positive assurance that the use of either would certainly cause "la locura"—*i. e.*, insanity or idiotcy.

There is, beyond any question, an abiding faith among Mexicans, shared, probably, by a large majority of them, a legendary, doubtless handed down through ages, that the properties of the "Marihuana" and of the "Toloache" are often invoked for the purposes of revenge; that juices, decoctions, or the seeds of these herbs are surreptitiously given to the intended victims with their food or drink, and that "la locura"—insanity or idiotcy—instantly follows. The writer has been cited a large number of such cases. Only a few days ago, in conversation upon this subject with a highly educated, refined gentleman, a firm believer in the fabulous properties of the "Toloache," he was given, as a case in point, that of a once wealthy and influential merchant of this city, who, because of refusal to credit an old Indian woman, lost his proud prestige by being reduced to idiotcy through the instrumentality of a dose of the juice of the "Toloache" leaves, which she secretly administered to him in a cup of coffee. Although reasoned, expostulated with and reminded that the fetid, narcotic odor, the bitter, nauseous taste of the Stramonium juice would preclude the possibility of such

*Vulgarly called "Jimson weed." †Also spelled "Mariguaua."

mode of administration, even if the quantity so taken was sufficient to produce the effects he described, he remained firm in the belief that the merchant's idiotcy was produced as detailed above. This gentleman is by no means alone in this opinion, for many, very many, of the inhabitants of Saltillo believe as he does; and so it goes, every manifestation of mental aberration, let the cause be what it may, is attributed to the effects of either Marihuana or Toloache. As a further illustration of this tendency, the three following examples are given :

A gentleman occupying high political and civic positions, universally respected and esteemed for his education and astuteness, relates that he left this city early one morning, bound for a neighboring village, in company with his "Mozo"; that about six o'clock in the morning, they halted at a ranch for refreshments, where his servant was given Toloache juice in his coffee; that violent mania ensued in a little while, which, after a month or two of extreme violence, gradually degenerated into idiotcy, from which he never recovered.

To the interrogative: How do you know your servant was given "Toloache" juice, he replied: "Because he was sound and well when we started on our journey, and became violently insane in about four hours after leaving the ranch where he ate his breakfast."

When asked: What motive could the "rancheros" have had in poisoning his servant, he responded; "None, other than a desire to do evil."

The late Dr. Gonzales, of Monterey, a man of extensive reputation, a little god among his people, said to have been a most excellent physician, an acute and an accurate observer, relates: (Lecciones de Materia Mèdica y Terepèutica.) "I will here add, that I have seen a very notable phenomenon in those poisoned by stramonium, of which no author makes mention. It is, that many, after the symptoms of grave (acute) poisoning disappear, remain for a long time, two or three years and more, stupid, indifferent and tactiturn." "These," he continues, "the people call *entoloachados*—*i. e.*, crazy from being poisoned by Toloache." The doctor then reports the following case: "There lived here a famous highwayman, a big thief and assassin, as bold as he was cunning, to whom his wife, because of maltreatment, administered Toloache seed in his tortillas." "The seeds," the doctor says, "were given for five or six days, in gradually increasing quantities, until they produced terrible poisoning, followed by almost complete idiotcy, from which the wretched victim was only released by death, sixteen years afterwards, although every method promising hope for his mental restoration had been tried."

With no desire to detract from the glorious immortelles that cluster around the memory of this good and universally beloved man, the writer strongly suspects that the doctor's observations and conclusions, as narrated above, were, in great part, if not in whole, *determined by a pre-existing belief*, rather than by the facts and probabilities that his cases presented. Indeed, this suspicion approximates quite nearly to a demonstrated fact, when it is remembered that he reports that a "bold" and "cunning man" ate, unaware, with his bread, enough of the bitter, acrid, nauseous stramonium seed, in five or six days, to reduce him to a condition of hopeless idiotcy, unless it be that the gustative functions of the glosso-pharyngeal, and the lingual branch of the fifth pair of nerves were greatly impaired, in which event—since it is well known that such manifestations are not infrequent precursors of violent maniacal outbursts—it is more than probable, certainly more in keeping with the observations of scientific investigators, that the "*primum mobile*" of the mental condition reported by Dr. Gonzales, preceded, instead of followed, the eating of the stramonium seed, and made the latter possible.

Sometime during last summer, the writer was called to see the wife of an Indian "del pueblo," reputed to have been "embrujado"—*i. e.*, bewitched by "Toloache." The following history was detailed him, with much precision: "One week ago la Señora returned home unexpectedly, and found her husband sharing the nuptial bed with another woman. A violent war of words, of fists, and of hair pulling succeeded, in which her outraged majesty was considerably punished. On the ensuing morning, a reconciliation was brought about by mutual friends, and with it the inevitable 'baile' and 'jarra de pulque.' With this 'distraccion' and this 'balm in Gilead,' all sins were washed away, all sorrows were drowned, and, if the stories of sorrowing and sympathetic friends can be believed, her ladyship's reason, too; for during the merrymaking incident to the pacification, the offending husband is charged with giving his wife to drink of 'pulque,' in which powdered seeds of the dreadful 'Yerba loca' had been concealed, whereupon she straightway became a victim, doomed to all the horrors of a mind bereft of reason."

This woman had been previously seen and treated by other physicians, one of whom—perhaps all—gave credence to the tale, and regarded the mental and physical phenomena, soon to be detailed, as having been caused by the "Toloache."

When first seen, this patient was sitting erect in a chair, as immobile as a piece of statuary. She had remained in this position three days and nights, voiding large quantities of limpid urine, expectorating an abundance of white, viscid, frothy saliva; without sleeping, without speaking, and without partaking of nourishment of any kind. Her pupils were widely dilated; breathing quick, shallow, irregular, and attended with an occasional long, deep inspiratory and expiratory sigh; pulse, rythm good, rate accelerated thirty beats; temperature 99½; lower portion of body, feet and legs gave no response to pricking with a needle; upper extremities did. There had been no weeping, no screaming, no convulsions nor local spasms, neither had there been a period of excitement, such as is caused by stramonium, that would not have been produced by the large quantity of "pulque" she had drunk.

Regarding the case as one of hysteria, the patient was treated with one-third grain of sulphate of morphia and one-hundredth grain of sulphate of atropia, hypodermically. She was placed in bed—by force—chloroformed, and a large enema of salts and senna administered—the latter completely unloading her bowels in a little while. She slept well the entire night, arose early, ate a hearty breakfast, and when seen, at 9 o'clock, was as rational and as happy as if discord had never embittered the sweetness of her married life.

It is presumed that but few, if any, will question the accuracy of the diagnosis recorded above, and just why the physicians, who saw the patient previously, all well instructed medical gentlemen, should overlook the wife's resentment of her husband's infidelity, the chagrin and mental excitement brought about by the encounter with her rival, the psychical condition incident to the reconciliation, and finally, the orgies with which that reconciliation was celebrated, in their search for the "casus morbi" of her condition and find it in the "Toloache" seeds which the Indians believed her husband had given her, unless there was *that pre-existing belief* in their minds, already assumed to be a sort of inheritance among this people, is, by no means, apparent to the writer; but that the untutored, superstitious aborigines should have done so, is not surprising.

If the current report of the day be a faithful reflex of the vicious use of the Indian hemp in the early days of Mexico, then the vice was widespread and general. It is said to be an historical fact, but the writer is unable to cite the work, that Axayacatl, father of Montezuma the Second, observing the prevalent abuse of "Mariguana," and its attending drunkenness among the Aztecs, ordered that every person found under its influence should be put to death; and that in obedience to this edict, more than twenty thousand victims were sacrificed in the city of Mexico within one week. Whether this be true or not, its abuse is not entirely suppressed even at the present day. Officers of the army are greatly annoyed by "Mariguana" smoking soldiers, who, according to military law, are punished with double the severity inflicted upon those found drunk from alcoholics.

That the long continued and immoderate use—just as might reasonably be expected from intoxicants and other narcotics—of either of the plants included in the term of "Yerba loca," is calculated to produce mental decay, no one acquainted with their properties will deny, but that either of them, singly or combined, was ever used with this end in view, and to its consummation, or that either, any, or all of them, ever produced such a result by another mode than that indicated, seems to rest on no better foundation than legendary, credulity and fabrication,—*i. e.*, the "Yerba loca," because of the mental disturbances produced by the plants entering into its make-up, has been a sort of receptacle among Mexicans, into which they vaguely cast every abnormal mental phenomena occurring among them.(*) This assertion is made after mature deliberation and patient investigation, and with full consciousness of the generally accepted fact, that the influences of many drugs are greatly modified by race, climate and condition.

To the professional mind, then, the facts contained in the preceding paragraphs, considered in conjuction with the further fact—the well-known intoxicating and narcotic properties of the plants, when taken in sufficient quantities, being remembered—that an extremely impressionable, highly superstitious, and an easily excited and credulous vein, permeates, and has, for all ages, permeated every sort and condition of this people, ready solutions for the many sensational stories about the so-called "Yerba loca" will present themselves.

SALTILLO, MEXICO, July 25, 1888.

### HEMORRHOIDS, FISSURES AND FISTULA IN ANO, AND THEIR TREATMENT.

By T. J. BENNETT, M. D., AUSTIN, TEXAS.

Read at Austin District Medical Society, June 13, 1888.

I HAVE been somewhat at a loss to know how I could present the three subjects of this paper so as to get the main points before the Society and yet remain within due limits as to the time allotted for papers to be read.

*An American missionary in this country reported having met, in some of his peregrinations, with a case of imbecility due to smoking Indian hemp, but on getting him to investigate the family history of the supposed victim, it turned out that the young man and his mother were epileptics ; hence it is concluded that the instability of the young man's nervous system, the insane diathesis under which he labored, provoked the vice, and that his mental decay was caused by the "epileptic habit," instead of by "Mariguana" smoking.

